# Study on Low-Temperature Performance Decay of Composite-Modified Porous Asphalt Mixture under Medium- and High-Temperature Water Erosion

**DOI:** 10.3390/ma16227079

**Published:** 2023-11-08

**Authors:** Chao Chai, Da Zhang, Zhongkun Wang, Guangya Ding

**Affiliations:** 1 Department of Civil Engineering, College of Architecture and Engineering, Wenzhou University, Wenzhou 325035, China; chaichao18@mails.jlu.edu.cn (C.C.); 00151050@wzu.edu.cn (G.D.); 2 Key Laboratory of Soft Soil Foundation and Coastal Reclamation Engineering Technology in Zhejiang Province, Wenzhou 325035, China; 3 Wenzhou Building Energy Conservation, Emission Reduction and Disaster Prevention and Reduction Engineering Technology Research Center, Wenzhou 325035, China; 4 Jiangsu Xiandai Road and Bridge Co., Ltd., Nanjing 210046, China; 5 Collaborative Innovation Center for mudflat Renovation Project and Ecological Protection in Zhejiang Province, Wenzhou 325035, China

**Keywords:** porous asphalt mixture, low-temperature performance, water erosion, acoustic emission

## Abstract

This paper studies the decay law of low-temperature crack resistance performance of rubber powder basalt fiber composite-modified porous asphalt concrete (CM-PAC) under medium- and high-temperature water erosion. Firstly, the prepared Marshall specimens were subjected to water erosion treatment at different temperatures of 20 °C, 40 °C, and 60 °C for 0–15 days. Then, the processed specimens were subjected to low-temperature splitting tests, and acoustic emission data during the splitting test process were collected using an acoustic emission device. It can be seen that the low-temperature splitting strength and low-temperature splitting stiffness modulus of CM-PAC gradually decrease with the increase in water erosion time. The maximum reduction rates of the two compared to the control group reached 72.63% and 91.60%, respectively. The low-temperature splitting failure strain gradually increases. Under the same erosion time, the higher the temperature of water, the more significant the amplitude of changes in the above parameters. In addition, it is shown that as the water erosion time increases, the first stage of loading on the specimen gradually shortens, and the second and third stages gradually advance. As the water temperature increases and the water erosion time prolongs, the acoustic emission energy released by the CM-PAC specimen during the splitting process slightly decreases. The application of acoustic emission technology in the splitting process can clarify the changes in the failure pattern of CM-PAC specimens during the entire loading stage, which can better reveal the impact of medium- to high-temperature water on the performance degradation of CM-PAC.

## 1. Introduction

Porous asphalt mixtures have been increasingly studied in recent years due to their advantages of rapid drainage and noise reduction [1,2,3]. They have a larger air void usually not less than 18% compared to dense-graded asphalt mixtures with an air void of only 3–6%. Rainwater is mainly discharged from the pavement structure through vertical and horizontal seepage inside the mixture, and water will have a scouring effect on the interior of the porous asphalt mixture. Therefore, porous asphalt mixtures require sufficient water stability. Many scholars have also conducted research on this aspect. Chen et al. [4] conducted a study on the water stability of double-layer porous pavement. The results indicated that the application of appropriate amounts of high-visibility modifier and polyolefin in double-layer porous pavement materials can improve water stability. In order to reduce road surface temperature and alleviate the urban heat island effect, Geng et al. [5] added cement mortar super-absorbent polymer to the porous asphalt mixture, and the experimental results revealed that the water-retaining asphalt mixture has good water stability, with a residual stability of not less than 88.2%. The above studies are all aimed at improving the water stability of the mixture through modification methods. In addition, it can be found from the following research that adding rubber powder, high-viscosity modifiers, polyurethane, and other materials to porous asphalt mixtures can effectively improve water stability [6,7,8,9]. Scholars have also studied the influence of water on the various properties of the mixture. Sapkota et al. [10] produced asphalt mixtures using recycled aggregates and found that the water stability was better than traditional asphalt mixtures. Their research findings provide a research foundation for the selection of pavement materials in humid environments. Ameri et al. [11] studied the antistripping ability of different types of porous asphalt mixtures under multiple water freeze–thaw cycles. It was showed that hydrated lime improves stability and durability of the mixture more than limestone powder. In the above studies, researchers have mostly used the immersion Marshall test and freeze–thaw splitting test methods. These two methods can quickly evaluate the water stability of several mixtures. However, they are not completely the same as the actual water damage process of porous asphalt mixtures. In the actual service process, the water damage to porous asphalt mixture is slow and long-term, and the mixture will be corroded by water at different temperatures. Further research is needed to evaluate the long-term water stability of the mixture through more practical testing methods.

Low-temperature crack resistance has always been a focus of research on the performance of asphalt mixtures, especially for asphalt materials in seasonally frozen areas. Wang et al. [12] studied the effects of air void, modifier content, aging conditions, and test temperature on the low-temperature performance of porous asphalt mixtures through low-temperature bending tests. It can be seen that modifier content is the most important factor in the low-temperature performance of porous asphalt mixtures, followed by test temperature, with porosity and aging conditions being the smallest. Some researchers have found that adding polymer substances, fiber modifiers, and the mineral oil-based rejuvenating agent to the mixture can effectively improve low-temperature crack resistance [13,14,15,16]. Furthermore, some researchers have optimized the design of the mixture based on low-temperature performance. Xu et al. [17] applied the Marshall design method to conduct freeze–thaw splitting tests on porous asphalt mixtures with limestone as aggregate. The test results showed that increasing the compaction temperature of the specimen can enhance the freeze–thaw splitting strength, while excessively increasing the compaction frequency of the specimen will lower the freeze–thaw splitting strength. Hu et al. [18] optimized the design of a double-layer porous asphalt mixture by considering parameters such as high- and low-temperature performance, and ultimately confirmed the optimal bitumen–aggregate ratio of the double-layer porous asphalt mixture. Li et al. [19] studied the crack resistance performance of epoxy porous asphalt mixtures through the Overlay Test. The research results indicated that the crack resistance of EPA13 is greatly affected by temperature. The above research focuses more on the low-temperature crack resistance performance indicators, and explores how to improve the low-temperature crack resistance performance of the mixture by adding various additives such as high-viscosity modifiers and fibers. However, the attenuation law of its low-temperature crack resistance performance after long-term water erosion is rarely considered.

With the continuous development of technological means, more and more new technologies are being applied to the research of asphalt mixtures [20,21,22]. As a new testing technology applied in the field of asphalt mixtures in recent years, acoustic emission technology can collect sound waves generated during asphalt mixtures’ deformation and damage, so this technology can be used to identify the damage situation of asphalt mixtures under load and other factors. Fu et al. [23] applied acoustic emission technology to study the crack resistance performance of different fiber asphalt mixtures. Through analysis of acoustic emission parameters, it can be concluded that steel fiber-reinforced asphalt mixtures exhibited more favorable crack resistance than glass fiber-reinforced asphalt mixtures and basalt fiber-reinforced asphalt mixtures. The relationship between crack evolution and acoustic emission parameters activity of fiber-emulsified asphalt cold-recycled mixtures was studied by Kong’s research, which reflected the microscopic failure mechanism of the material [24]. Behnia [25] evaluated the low-temperature crack resistance performance of five asphalt mixtures using acoustic emission technology parameters. The results showed that this technique is very sensitive to aging level of asphalt materials as well as the presence of additives such as warm-mix asphalt mixture additives within binder or mixture. Qiu et al. [26] recorded the acoustic emission signals in the three-point bending test of asphalt mixtures and conducted clustering analysis on the signals using the k-means method, ultimately establishing the relationship between parameter characteristics and damage behavior. Wei et al. [27] monitored the uniaxial compression test process of asphalt mixtures under six loading rates using acoustic emission technology, and the test results showed that the failure mode of asphalt mixtures under different loading rates can be identified according to the correlation difference of acoustic emission parameters and the difference in dominant-frequency evolution distribution. Liang et al. [28] studied the high-temperature compressive and low-temperature crack resistance of asphalt mixtures after freeze–thaw cycles, and verified that acoustic emission technology can effectively reflect the failure characteristics of specimens at high and low temperatures. Based on the above research, it can be concluded that acoustic emission technology can effectively characterize the failure process of asphalt mixtures under various forms of load.

All in all, in order to break through the limitations of current standard methods for testing the water stability of porous asphalt mixtures, this article focuses on studying the low-temperature performance changes of CM-PAC after medium- to high-temperature water erosion. We propose a new experimental method. Firstly, the CM-PAC Marshall specimens were treated with different water temperatures and erosion times through indoor experiments. Then, the acoustic emission technology was applied to the mechanical test of low-temperature splitting, and the acoustic emission signals of the specimens in the low-temperature splitting test were collected. Finally, the internal damage pattern of CM-PAC after medium- to high-temperature water erosion was further clarified through the analysis of experimental data.

## 2. Materials and Methods

### 2.1. Raw Materials

We used steel slag as aggregate in this study, which was produced by Jilin Dongsheng company in Jilin, China. We used limestone powder as filler, which was also produced by Jilin Dongsheng company in Jilin, China. Their main physical and mechanical properties are shown in Table 1. The binder used in this study was SBS-modified bitumen. It was produced in Panjin, China. The amount of SBS modifier was 4% and its main indicators are shown in Table 2. In addition, we used the response surface method to determine the optimal dosage of three materials, obtained from a previous study [29]. Specifically, the asphalt to aggregate ratio, crumb rubber content, and basalt fiber content were set as independent variables, and the air void, Marshall stability, flow value, Marshall quotient, and Cantabro particle loss were set as the response values. Through model analysis, the optimal amount of rubber powder used was 11.21%, and the optimal amount of basalt fiber used was 0.42%. The optimal bitumen–aggregate ratio was 4.51%. The specimens in this study continued to use the above material dosage.

### 2.2. Sample Processing

Firstly, 48 standard Marshall specimens were manufactured according to the standard test methods in the specifications [30]. Then, the specimens were divided into three groups and subjected to water bath erosion at different times and at 20 °C, 40 °C, and 60 °C. The water erosion at 20 °C simulated the effect of ambient-temperature water on porous asphalt mixtures. Water erosion at 40–60 °C simulated the effect of high-temperature weather water on porous asphalt mixtures. Finally, the corroded specimens were stored at −10 °C and the splitting test completed. Each group had three parallel specimens. The sample processing is shown in Table 3.

### 2.3. Low-Temperature Splitting Test and Acoustic Emission Parameters

**Figure 1:**
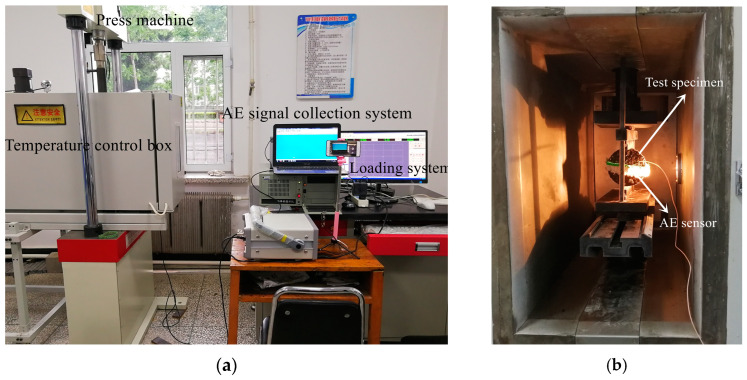
Low-temperature splitting test. (**a**) Low-temperature splitting test loading system; (**b**) internal diagram of the incubator.

The main parameters of acoustic emissions include energy, amplitude, ringing count, etc. Energy is the size of the area enclosed by the acoustic emission signal curve and the time axis, directly characterizing the strength of the acoustic emission-event activity, and is a representative indicator for evaluating material damage. The low-temperature splitting test temperature was −10 °C. All Marshall specimens were insulated in a temperature-control box for 5 h before the start of the experiment. First, an appropriate coupling agent was evenly applied on the surface of the acoustic emission sensor, and then an elastic band was used to stabilize the sensor to the surface of the test piece. After confirming that the coupling between the sensor and the surface of the specimen was intact, the testing machine was started and loaded at a uniform rate of 1 mm/min. After the test piece was damaged, the test was stopped and the data saved. Figure 1 shows the test site. Based on the test results and according to T 0716-2011 and T 0713-2000 in the specifications [30], the calculation methods are shown in Equations (1)–(3).

(1) $$R_{T}=0.006287P_{T}/h$$

(2) $$\varepsilon_{T}=X_{T}(0.0307+0.0936\mu )/(1.35+5\mu )$$

(3) $$S_{T}=P_{T}\times (0.27+1.0\mu )/(h\times X_{T})$$

where *R_T_* is the splitting strength, *P_T_* is the maximum force in the splitting test, *h* is the height of the specimen, *X_T_* is the deformation in the horizontal direction, *ε_T_* is the splitting–failure strain, and *S_T_* is the splitting stiffness modulus.

## 3. Results and Discussion

### 3.1. Analysis of Low-Temperature Splitting Test Parameters

#### 3.1.1. Low-Temperature Splitting Strength

**Figure 2:**
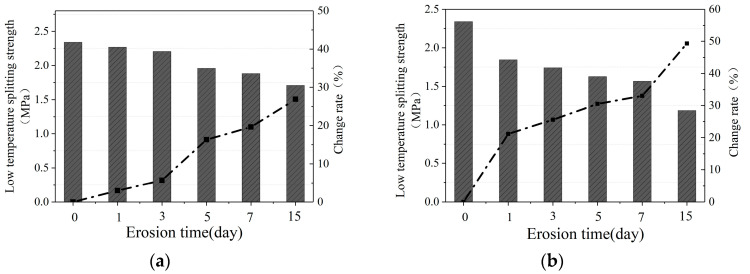

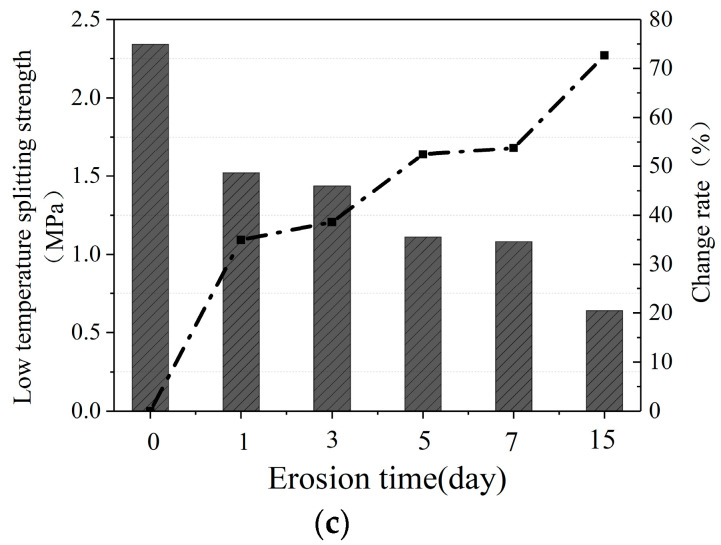
Low-temperature splitting strength of PACs. (**a**) 20 °C water erosion; (**b**) 40 °C water erosion; (**c**) 60 °C water erosion.

Figure 2a–c show the low-temperature-splitting strength of the mixture after water erosion at 20 °C, 40 °C, and 60 °C, respectively. The group with a water erosion time of 0 in the figure is the control group, which is not affected by water erosion. In addition, we use the “change rate” to represent the changes in relevant parameters after water erosion compared to the control group parameters, represented by black dashed lines in the figures.

It can be seen that after water erosion at different temperatures, the low-temperature splitting strength of CM-PAC gradually decreases with the increase in water erosion time. Under the erosion of water at 20 °C, after 1, 3, 5, 7, and 15 days, the low-temperature splitting strength of CM-PAC decreased by 3.01%, 5.72%, 16.30%, 19.61%, and 27.00%, respectively. Under the erosion of water at 40 °C, after 1, 3, 5, 7, and 15 days, the low-temperature splitting strength of CM-PAC decreased by 21.18%, 25.60%, 30.49%, 33.00%, and 49.30%, respectively. Under the erosion of water at 60 °C, after 1, 3, 5, 7, and 15 days, the low-temperature splitting strength of CM-PAC decreased by 34.99%, 38.60%, 52.46%, 53.73%, and 72.63%, respectively.

In addition, under the same water erosion time conditions, the higher the water temperature, the more significant the decrease in the strength of CM-PAC. For example, after 1, 3, 5, 7, and 15 days of water erosion, the reduction rate of splitting strength caused by 60 °C water is 11.64, 6.75, 3.22, 2.74, and 2.69 times that caused by 20 °C water, respectively. It can also be seen that as the water erosion temperature increases, the rate of decrease in low-temperature splitting strength gradually slows down. This indicates that the low-temperature splitting strength of CM-PAC does not linearly decrease with the increase in water erosion temperature.

#### 3.1.2. Low-Temperature Splitting Failure Strain

**Figure 3:**
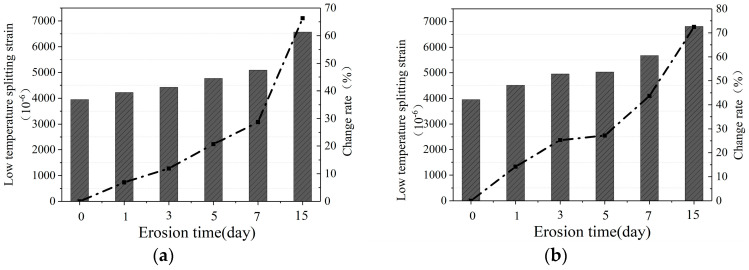

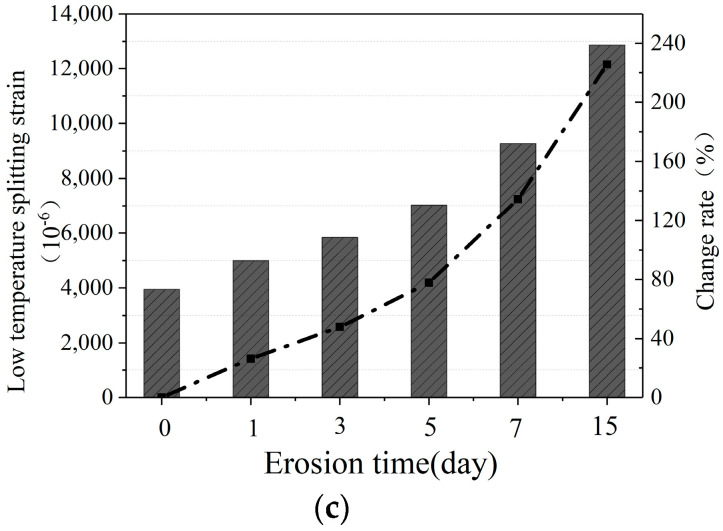
Low-temperature splitting failure strain. (**a**) 20 °C water erosion; (**b**) 40 °C water erosion; (**c**) 60 °C water erosion.

Figure 3a–c show the low-temperature splitting failure strain of the mixture after water erosion at 20 °C, 40 °C, and 60 °C, respectively.

It can be seen from Figure 3 that after water erosion at different temperatures, the low-temperature splitting failure strain of CM-PAC gradually increases with the increase in water erosion time. Under the erosion of water at 20 °C, after 1, 3, 5, 7, and 15 days, the low-temperature splitting failure strain of CM-PAC increased by 6.90%, 11.88%, 20.69%, 28.74%, and 66.28% compared to the control group, respectively. Under the erosion of water at 40 °C, the low-temperature splitting failure strain of CM-PAC increased by 14.18%, 25.29%, 27.20%, 43.68%, and 72.41% compared to the control group, respectively. Under the erosion of water at 60 °C, the low-temperature splitting failure strain of CM-PAC increased by 26.44%, 47.89%, 77.78%, 134.48%, and 225.67% compared to the control group, respectively. Similar to the pattern of changes in low-temperature splitting strength indicators, the higher the eroded water temperature, the more significant the increase in splitting failure strain. For example, after 1, 3, 5, 7, and 15 days of erosion, the increase rate of failure strain caused by 60 °C water is 3.83, 4.03, 3.76, 4.68, and 3.40 times that of the increase rate caused by 20 °C water, respectively. During the process of water erosion of porous-asphalt mixtures, higher water temperatures can cause the surface of the mixture to peel off and the interior to loosen.

#### 3.1.3. Low-Temperature Splitting Stiffness Modulus

**Figure 4:**
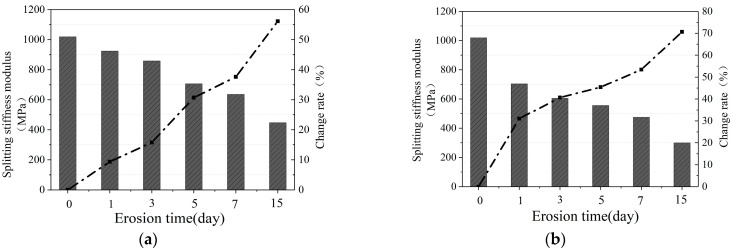

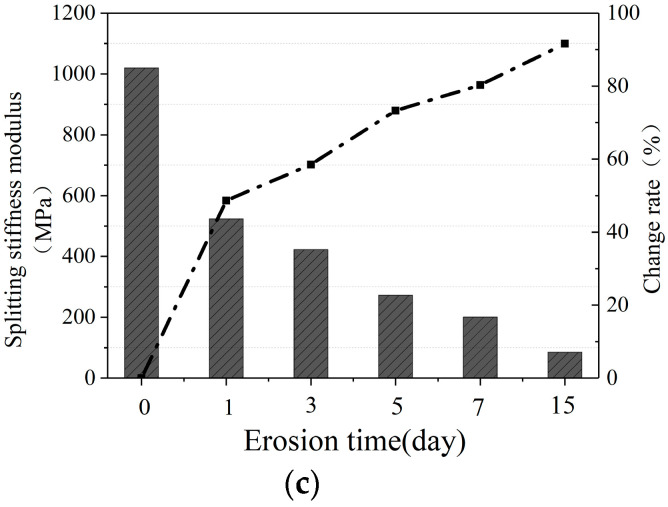
Low-temperature splitting failure stiffness modulus. (**a**) 20 °C water erosion; (**b**) 40 °C water erosion; (**c**) 60 °C water erosion.

Figure 4a–c show the low-temperature splitting stiffness modulus of the mixture after water erosion at 20 °C, 40 °C, and 60 °C, respectively.

It can be seen that, after water erosion at different temperatures, the low-temperature splitting stiffness modulus of CM-PAC gradually decreases with the increase in water erosion time. Under the erosion of water at 20 °C, after 1, 3, 5, 7, and 15 days, the low-temperature splitting stiffness modulus of CM-PAC decreased by 9.34%, 15.80%, 30.71%, 37.61%, and 56.13%, respectively. Under the erosion of water at 40 °C, the low-temperature splitting stiffness modulus of CM-PAC decreased by 30.03%, 40.66%, 45.40%, 53.41%, and 70.62%, respectively. Under the erosion of water at 60 °C, the low-temperature splitting stiffness modulus of CM-PAC decreased by 48.63%, 58.52%, 73.28%, 80.28%, and 91.60%, respectively.

In addition, similar to the law of low-temperature splitting strength attenuation, the higher the temperature of water, the more significant the decrease in splitting stiffness modulus values, and the more severe the erosion effect on CM-PAC. Under water erosion at 60 °C, the rate of decrease in low-temperature splitting stiffness modulus gradually slows down. This indicates that the low-temperature splitting stiffness modulus of CM-PAC does not exhibit linear decay with the prolongation of high-temperature water erosion time. This is because high-temperature water has caused significant damage to the specimen in a short period of time, and the specimen has significantly softened.

From the microscopic perspective of matter, temperature is an important factor affecting the activity of water molecules. The higher the temperature, the more active the water molecules are, making it easier for them to invade the interface between the asphalt film and the aggregate, thereby making the mixture looser. Therefore, higher temperatures of water will make the asphalt mixture more softened, with lower strength, leading to greater strain changes. It can be inferred that in hot summers precipitation will cause greater damage to permeable pavement materials.

### 3.2. Analysis of Acoustic Emission Parameters of CM-PAC

**Figure 5:**
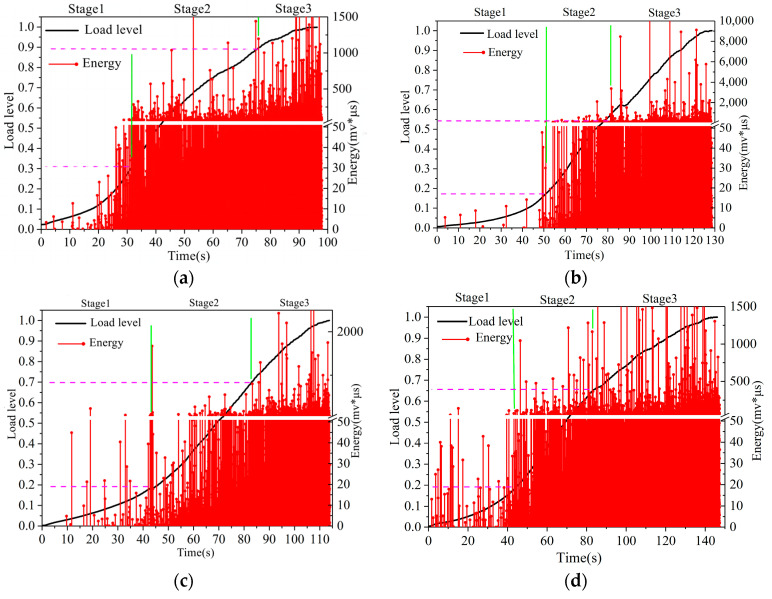

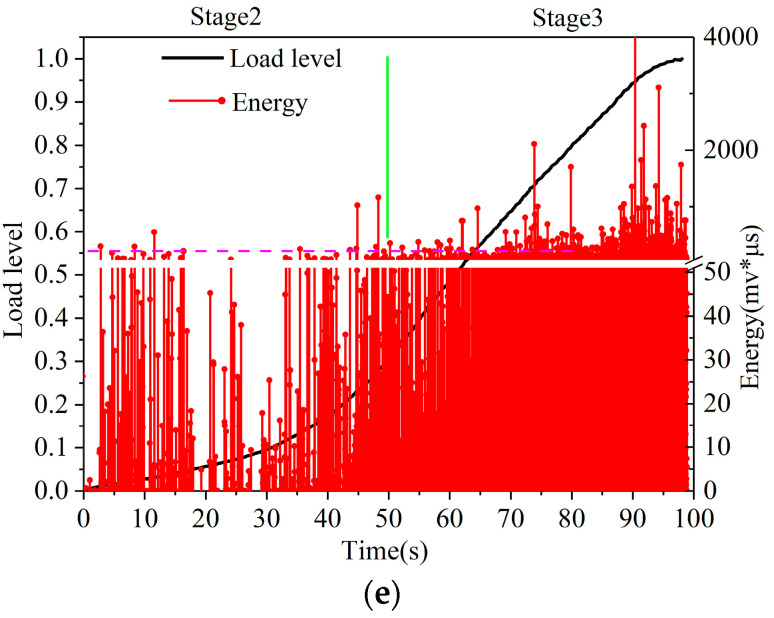
“Energy-load level” diagram of CM-PAC under water erosion at 20 °C. (**a**) 1 day; (**b**) 3 days; (**c**) 5 days; (**d**) 7 days; (**e**) 15 days.

**Figure 6:**
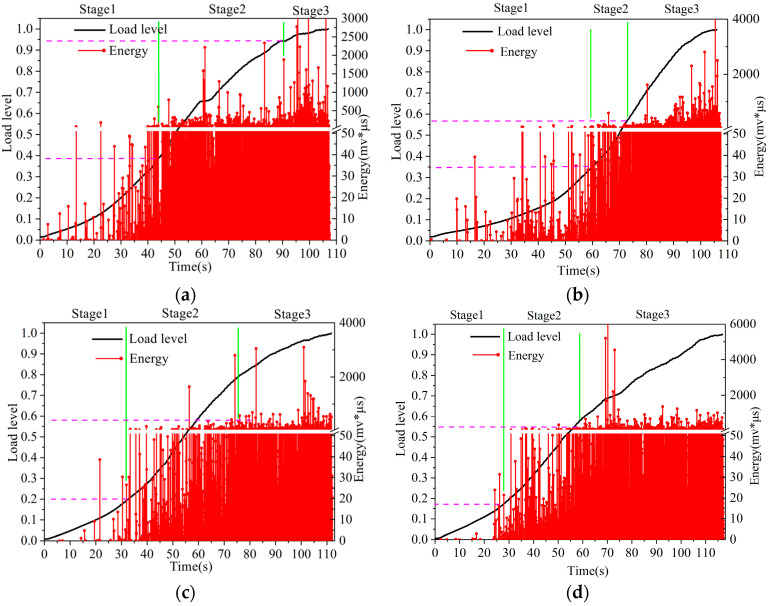

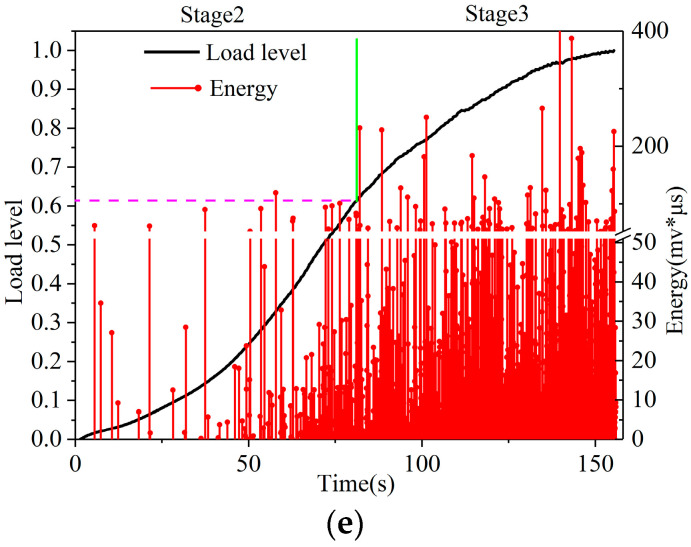
“Energy-load level” diagram of CM-PAC under water erosion at 40 °C. (**a**) 1 day; (**b**) 3 days; (**c**) 5 days; (**d**) 7 days; (**e**) 15 days.

**Figure 7:**
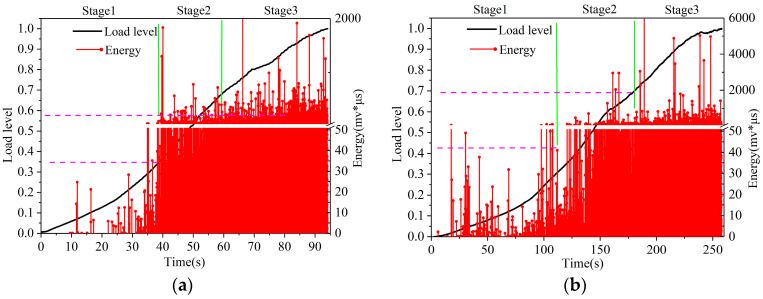

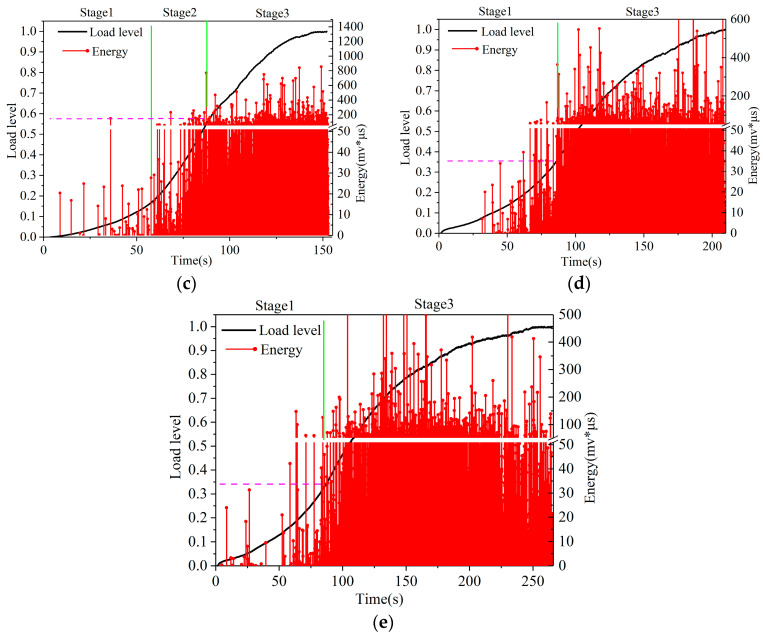
“Energy-load level” diagram of CM-PAC under water erosion at 60 °C. (**a**) 1 day; (**b**) 3 days; (**c**) 5 days; (**d**) 7 days; (**e**) 15 days.

In order to further analyze the failure process of CM-PAC and clarify the impact of medium- to high-temperature water on the crack resistance performance of CM-PAC, the acoustic emission–energy parameters and the normalized load are shown in Figure 5, Figure 6 and Figure 7.

Figure 5 shows the “energy-load level” diagram of CM-PAC based on a low-temperature splitting test after water erosion at 20 °C. The horizontal axis represents the time of the splitting test, while the vertical axis represents the load level and energy value. According to the load level and the energy density in the acoustic emission parameters, the entire process can be divided into two to three stages. The first stage is from the beginning of loading until the load level reaches around 0.3. During this stage, a small amount of intermittent acoustic emission signals appears, corresponding to the compaction stage of the loading process of the specimen. The test piece undergoes certain deformations and the internal mixture particles experience relative slip. At the same time, some small cracks and damages may have occurred at the weak points of the specimen. In the second stage, the acoustic emission signal values that appear become obviously denser and the energy values also increase clearly. This indicates that during this stage, the specimen releases apparent energy, resulting in significant damage, and the small cracks generated in the first stage further expand. Finally, as the loading continues and reaches the third stage, the specimen enters the failure stage. At this point, the frequency of the acoustic emission signal reaches its maximum and the energy value also reaches its maximum. The specimen undergoes prominent deformation, with internal cracks further expanding until the specimen is destroyed.

Furthermore, by comparing Figure 5a–e, it can be seen that with the increase in water erosion time, the energy parameters in acoustic emissions exhibit a certain pattern. For specimens with shorter water erosion time, as shown in Figure 5a,b, the signal values in the first stage are very low, indicating that the damage degree of the specimen is very small during the initial loading stage. When the water erosion time continues to increase, as shown in Figure 5c,d, the signal values in the first stage proportionately increase, indicating that after a long period of water erosion, damage occurs inside the specimen, and significant energy release occurs in the early stages of loading. When the water erosion time increases to 15 days, as shown in Figure 5e, the specimen exhibits dense acoustic emission signal values in the early stage of loading, and the energy amplitude is relatively large. The specimen directly entered the second stage. This indicates that after a long period of room-temperature water erosion, remarkable damage has occurred inside the CM-PAC. Under the action of load, there is immediately measurable energy release inside the mixture, and the specimen is prone to cracking.

Figure 6 shows the “energy-load level” diagram of CM-PAC based on low-temperature splitting test after water erosion at 40 °C. Similar to the pattern reflected in Figure 5, the loading process can still be divided into three stages. In addition, it can be observed that as the water erosion time increases, the first stage gradually shortens, and the second and third stages gradually advance.

Figure 7 shows the “energy-load level” diagram of the CM-PAC specimen under water erosion at 60 °C based on the low-temperature splitting test, which shows a similar pattern to Figure 5 and Figure 6. That is, as the water erosion time increases, the first stage gradually shortens, and the second and third stages gradually advance. This indicates that after long-term erosion by high-temperature water, noticeable damage has occurred inside the specimen, and under the action of splitting load the crack propagation and failure stages inside the specimen will be significantly advanced. For Figure 7d,e, it is worth noting that the maximum acoustic emission energy released during the loading process of the specimen comes from the middle stage of loading, indicating that the specimen has already developed obvious cracks at this time. After this, although it can still bear the load, it has already experienced significant damage. Therefore, we excluded the second stage. In addition, with the increase in water temperature and water erosion time, the energy value of the specimen during the splitting process slightly decreases. This is because the higher the water temperature and the longer the erosion time, the more severe the damage inside the CM-PAC, and the smaller the total potential energy inside it.

Through the above analysis and research, it can be concluded that the application of acoustic emission technology in the splitting process can clarify the damage process of CM-PAC under the action of medium- and high-temperature water erosion, and clarify the variation pattern of specimen failure during the entire splitting–loading stage.

## 4. Conclusions

This article conducted a study on the low-temperature performance decay of the optimized CM-PAC under the action of medium- to high-temperature water erosion. The effects of water erosion duration and water temperature on the mechanical properties of CM-PAC were analyzed through low-temperature splitting test results and combined with acoustic emission data, and the division of the damage stages was completed. The main conclusions were as follows:

(1) After water erosion at 20 °C, 40 °C, and 60 °C, the low-temperature splitting strength and low-temperature splitting stiffness modulus of CM-PAC gradually decrease with the increase in water erosion time, and the low-temperature splitting failure strain gradually increases.
(2) Under the same erosion time, the higher the temperature of water, the more significant the amplitude of changes in the above parameters.
(3) As the water erosion time increases, the internal crack propagation stage and failure stage of the specimen under load will dramatically advance.
(4) As the water temperature and water erosion time increase, the acoustic emission energy value of the CM-PAC specimen slightly decreases during the splitting process. The higher the water temperature, the longer the erosion time, and the more severe the damage inside the CM-PAC.
(5) The application of acoustic emission technology in the splitting process can identify the damage process of CM-PAC under medium- and high-temperature water erosion, and clarify the changes in the failure pattern of CM-PAC specimens during the entire splitting–loading stage. This better reveals the effect of medium- to high-temperature water on the degradation of CM-PAC performance.
(6) The conclusion of this study is based on indoor experimental results. It partly reveals the influence of water on the low-temperature crack resistance of pavement materials. However, for practical service pavements, both water volume and temperature are time-varying. Further research is needed to obtain more meaningful conclusions based on actual conditions.

## Figures and Tables

**Table 1:** Properties of aggregates and filler [29].

Index	Apparent Specific Density (g/cm^3^)	Los Angeles Abrasion (%)	Crushed Stone Value (%)
Coarse aggregate	3.527	12.9	13.9
Fine aggregate	3.389	-	-
Filler	2.722	-	-

**Table 2:** Properties of SBS-modified bitumen [29].

Properties	Results	Chinese Standard
Penetration (25 °C, 0.1 mm)	65.2	60–80
Softening point (°C)	64.2	≥55
Ductility (5 °C, cm)	34.5	≥30
Flash point (°C)	264	≥230
Elastic recovery (25 °C, %)	91.7	≥65

**Table 3:** Water erosion treatment of test pieces.

Erosion Duration (Days)	0	1	3	5	7	15	Temperature (°C)
Number of test pieces	3	3	3	3	3	3	20
		3	3	3	3	3	40
		3	3	3	3	3	60

## Data Availability

All relevant data are contained in the present manuscript.
